# The neonatal adverse event severity scale: current status, a stakeholders' assessment, and future perspectives

**DOI:** 10.3389/fped.2023.1340607

**Published:** 2024-01-08

**Authors:** Karel Allegaert, Thomas Salaets, Kelly Wade, Mary A. Short, Robert Ward, Kanwaljit Singh, Mark A. Turner, Jonathan M. Davis, Tamorah Lewis

**Affiliations:** ^1^ Department of Development and Regeneration, KU Leuven, Leuven, Belgium; ^2^ Department of Pharmaceutical and Pharmacological Sciences, KU Leuven, Leuven, Belgium; ^3^ Department of Clinical Pharmacy, Erasmus MC, Rotterdam, Netherlands; ^4^ Pediatric Cardiology, University Hospitals, Leuven, Belgium; ^5^ Department of Pediatrics, University of Pennsylvania Perelman School of Medicine, Philadelphia, PA, United States; ^6^ International Neonatal Consortium, Communications Workgroup, Tucson, AZ, United States; ^7^ Department of Pediatrics, University of Utah, Salt Lake City, UT, United States; ^8^ International Neonatal Consortium, Critical Path Institute, Tucson, AZ, United States; ^9^ Institute of Lifecourse and Medical Sciences, University of Liverpool, Liverpool Health Partners, Liverpool, United Kingdom; ^10^ Centre for Women’s Health Research, Liverpool Women’s Hospital, Liverpool, United Kingdom; ^11^ Department of Pediatrics, Tufts Children’s Hospital, Tufts University School of Medicine, Boston, MA, United States; ^12^ Department of Pediatrics, City School of Medicine, Kansas Children’s Mercy Hospital, University of Missouri Kansas, Kansas City, MO, United States; ^13^ Division of Clinical Pharmacology and Toxicology, Hospital for Sick Children, Toronto, ON, Canada

**Keywords:** newborn, infant, clinical trials, data standards, drug development, drug safety, adverse event

## Abstract

To support informed decisions on drug registration and prescription, clinical trials need tools to assess the efficacy and safety signals related to a given therapeutic intervention. Standardized assessment facilitates reproducibility of results. Furthermore, it enables weighted comparison between different interventions, instrumental to facilitate shared decisions. When focused on adverse events in clinical trials, tools are needed to assess seriousness, causality and severity. As part of such a toolbox, the international Neonatal Consortium (INC) developed a first version of the neonatal adverse event severity scale (NAESS). This version underwent subsequent validation in retro-and prospective trials to assess its applicability and impact on the inter-observer variability. Regulators, sponsors and academic researchers also reported on the use of the NAESS in regulatory documents, trial protocols and study reports. In this paper, we aim to report on the trajectory, current status and impact of the NAESS score, on how stakeholders within INC assess its relevance, and on perspectives to further develop this tool.

## 1 Introduction: the relevance of a valid adverse event severity scale in neonatal trials

Despite different legal incentives and initiatives, neonates are still commonly treated with medicines that have not been specifically labeled for this population. This is also reflected in the report provided in September 2022 by the Food and Drug Agency (FDA), when the historic milestone of 1,000 drugs or biologics having new pediatric use information in the label was published (1). Improved drug labelling for children was most pronounced for infectious diseases, psychiatry and dermatology, while changes in label information for neonates remained rare. The reasons why drug regulatory trials are more difficult in the “neonatal arena” are diverse, and include the still limited understanding of neonatal pathophysiology, the small market, as well as challenges related to trial conduct, including assessment of efficacy and drug safety.

To streamline drug development efforts and make the “neonatal arena” trial ready, the FDA and Critical Path Institute established the International Neonatal Consortium (INC) in 2015. INC brought together key stakeholders from parent advocates, nursing representatives, regulators, industry and the academic community from around the globe, with a focus on regulatory science and improved drug labelling in neonates. Among other interests, INC hereby intends to co-create tools to improve clinical trial efficiency and success, including tools to assess efficacy and safety of medicines in neonates (2).

Adverse events reporting in neonatal clinical trials is difficult. Tools are needed to assess seriousness, causality and severity. While seriousness has a clear regulatory definition, causality and severity have their issues. Discriminating patient confounders from dose related adverse drug reactions (ADR) in neonates remains a major challenge. The principal difference between an adverse event and ADR is that causality is at least suspected for the latter. While the regulatory environment on causality assessment and reporting in neonates is similar to other populations, its assessment in neonates is more difficult. This is in part due to inconsistent terminology and case description, further complicated because signal detection in a “noisy” setting with extensive variability in commonly used biomarkers, relevant and diverse morbidity characteristics, and the many comorbidity-related signals in this population (3, 4). A population specific tool (modification of the original Naranjo algorithm to neonates) to assess causality in neonates has been reported by Du et al. Based on 13 items (yes/no/not applicable), categorization of causality (definite, probable, possible, unlikely, not related) was facilitated (5). This modified Naranjo score was subsequently prospectively tested and compared to the Liverpool ADR Causality Assessment Tool and the Karch and Lasagna method in a dataset of suspected ADRs. Irrespective of the tool, and despite the fact that the study was conducted on a dataset of suspected ADRs, only “fair” inter-rater and inter-tool reliability were reached (6).

Severity assessment of adverse events is also challenging. Clinical trialists have attempted to use adult-specific AE severity scales for the neonatal population with varying success. To illustrate the challenge, imagine trying to interpret what “affecting activities of daily life”- a typical adult AE severity criterion—would mean for a newborn. Accurate reporting of AEs also necessitates an understandable common language that makes this information interpretable for all stakeholders. A standardized AE severity scale provides such a common language. Prior to 2019, such a scale to standardize AEs observed in clinical trials in neonates was not yet available.

## 2 Steps taken to develop the neonatal adverse event severity (NAESS) scale

In 2019, INC reported on the NAESS to standardize severity reporting in neonates (7). Following a modified Delphi approach with input from the different stakeholders involved, it contains diagnosis-specific severity grading criteria for a set of 35 typical and common neonatal AEs. Furthermore, the scale also has a generic neonatal AE severity grading table that uses criteria relevant to neonates to define severity of any other possible AE. Laboratory values were not considered in this initial tool, as reference or normal values are still poorly described, another area in need for further development (8).

The NAESS tool is also available under “INC Terminology” through the Thesaurus of the US National Cancer Institute, linked to the Medical Dictionary for Regulatory Activities (MedDRA) (9). This co-aligns with existing severity scales in other patient populations and research fields, while applying characteristics relevant to neonates. It is hereby embedded in and linked to other terminology sources used for other, but sometimes overlapping, purposes.

The NAESS scale is unique in that it consider changes the baseline clinical status of neonates as major criterion. This is particularly relevant to neonates admitted in neonatal intensive care unit (NICU)s, where many of the clinical trials are conducted. Although severity scales are typically consensus documents that intend to reduce inter-observer variability, follow up studies are needed to assess the impact on this variability. To document the impact of the NAESS tool on the inter-rater variability, both a retrospective and prospective study were conducted.

Using real-world data on 60 AEs previously collected from a neonatal trial, 12 randomly assigned reviewers assessed these events with a total of 240 severity scores. When reviewers applied either the generic or AE-specific NAESS. The intraclass correlation coefficient (ICC) of 0.63 reflected moderate reliability. Based on the retrospective design, the authors concluded that source data collection on the neonatal AE forms used in clinical trials can be improved and that augmented training on the NAESS tool was needed (10). In a follow-up prospective study, severity was assessed by two independent observers in each of 4 NICUs across the world, initially using a generic, non-specific scale, then subsequently in a second phase with the INC NAESS tool. Structured training on the use of the NAESS tool preceded the use of the tool in the second phase of the study. Based on 240 AEs assessed, ICC was significantly higher (0.69 compared to 0.66) in the second phase, most pronounced (ICC 0.80) for those AEs for which event-specific AE guidance was available (11).

## 3 Impact

Assessing the full uptake of the NAESS tool would necessitate a very extensive search of the grey literature. After advice of librarians of KU Leuven (Krizia Tuand, Thomas Vanderdriessche), we therefore opted for a pragmatic, explorative approach, with focus on regulators, trial uptake and its use by sponsors.

Related to regulators, we searched (September 2023) the FDA and the European Medicines Agency (EMA) website on guidance and guidelines related to NAESS (12). In the recently updated FDA guidance (July 2022), the NAESS tool is mentioned as a reference. In contrast, we understood that the EMA neonatal guidelines document will be updated in the next year(s), so that its current version (legal effective date 01.01.2010) does not yet contain any specific suggestion on severity assessment (13). During a revision of the relevant EMA guideline, the NAESS scale could be included.

Related to uptake by academia, we performed a citation tracking with snowball sampling on NAESS publications (October, 10th 2023) in PubMed, the journal's website and Google Scholar. Furthermore, we inquired within the INC network and beyond on the use of the NAESS tool in study protocols. We identified clinical trials protocols with NAESS that were related to intubation practices (video vs. direct laryngoscopy) (14), an artificial intelligence tool to detect adverse drug reactions based on severity and probability (15), fetal safety indicators, and its application for somatic cell gene therapies or fetal myelomeningocele repair (16–20), a pentoxifylline optimal dose finding trial (21), ripasudil eye drops (to prevent retinopathy of prematurity (22) or doxapram (to treat apnea of prematurity) trial (23, 24).

Within the INC network and its members, we were informed that there are other drug development programs related to neonatal nutrition, retinopathy of prematurity, and neonatal asphyxia that incorporated the NAESS tool in their study protocol. We also became aware of one consulting activity (related to a perinatal clinical trial development plan on tocolysis), in whom the sponsor considered the NAESS as part of efficacy outcome variables. However, we would like to stress that the tool is rather developed as an AE severity tool, likely less suited as an efficacy variable (*indications are mentioned, specific compounds not discussed to respect confidentiality*).

## 4 Stakeholders' reflections on the NAESS scale

The multistakeholder perspective is a specific strength of the INC consortium, bringing together parents, nurses, regulators, industry, and clinicians/academia. INC conducted a multistakeholder survey (parents, nurses, and neonatologists) to obtain perspectives of research-related education and communication practices in the NICU. Differences were noted with respect to unmet medical needs of sick neonates, research mission of the NICU, education/training the research team, and research communication provided to parents. Opportunities identified were engagement of nurses and parents at all stages of NICU research, and education on the research process and protections for all stakeholders (25).

To address the survey findings, INC Communication Work Group has focused on the development of key messages for the projects within INC. This group hereby applies the guidance provided by the Model Systems Knowledge Translation Center (MSKTC) as a tool for consistency on purpose, format, audience and resources when developing key messages from and to the different stakeholders (26). Specific for the NAESS, this group brought together the different stakeholders to reflect on the relevance of the current NAESS version and on future perspectives shortly after initial development (2019), and once the validation efforts were reported (2023). We here summarize the key messages of the different stakeholders throughout this process, as it is relevant to be aware of similarities, as well as specific interests of the different stakeholders involved. Understanding multistakeholder perspectives can provide opportunities to optimize future neonatal clinical trials (Table 1).

**Table 1:** Stakeholder specific key messages related to the neonatal adverse event severity scale (NAESS) tool.

Stakeholder	Stakeholder specific key messages
Parents	The scaling of the NAESS model clarifies safety reporting criteria, reducing subjectivity in severity assessments and increasing the safety of each baby enrolled in a clinical trial. Parents may recognize a change in their infant's baseline condition. Support the parents in learning about adverse events and identifying when to raise a red flag. Understanding adverse events may empower parents to reduce the risk of the event's reoccurrence or mitigate its negative impact. Engaging parent participation in clinical care and clinical trial reporting of adverse events further increases the overall safety of all infants, including those enrolled in a clinical research trial.
Nurses	The use of the NAESS will lead to clear communication for AE identification and evaluation. The availability of an educational tool is helpful to increase inter-rater reliability. Application of nurses’ clinical expertise in AE identification and documentation is critical, and research nurses demonstrated high level of inter-rater reliability following its use. The NAESS will support nurses to concurrently identify changes in baseline to better identify the occurrence and severity of AEs, thereby contributing to the overall safety of clinical care and trials. Real time clinical assessment may serve to increase inter-rater reliability. NAESS, when used consistently by all stakeholders, including nurses, will strengthen the research culture shared by the multidisciplinary team.
Regulators	NAESS emphasizes that neonates are different and describes neonatal morbidity helping to differentiate disease and intervention-related events NAESS defines a better language, common to all parties involved in the care of the neonate and their enrollment in a study/trial ​ NAESS is aligned with MedDRA and NIH to meet regulatory needs for submissions. This provides a more complete picture of risk, benefit and causality assessment and informs pharmacovigilance, pediatric safety reports/summaries, benefit/risk discussion and labeling. Training to all health care personnel should be provided on the NAESS scale. This training could be extended to all those who care for the neonates/infants, including the parents and other caregivers to ensure that no adverse events of significance are missed.
Industry	INC NAESS addresses a current gap for the conduct of neonatal trials, as existing severity scales not appropriate for neonates. To do so, the NAESS scale was developed by a diverse set of global stakeholders to enhance the quality of the data and assist data safety monitoring boards, sponsors and regulators in safety reporting. We hereby collaborated with the Medical Dictionary for Regulatory Activities (MedDRA) to create specific NAESS adverse event terminology by mapping of the AEs to MedDRA Lowest Level Terms (LLTs). Following its design, validation efforts (retrospective, prospective) were performed, and a training module developed to facilitate the implementation of this tool to enhance safety of infants in clinical trials was made available. Infant safety is critically important in both clinical care and research. The NAESS is the first tool to address the lack of standardization in how safety events are assessed on severity. It provides a standardized, infant-specific framework to consistently assess severity of the most commonly reported events experienced among critically ill infants.
Clinicians	Parents, nurses, pharmacists, neonatologists, and clinical research team members all play important roles in recognizing changes to an infant's baseline condition, identifying possible AEs, and promoting a safety culture Establishing tools that promote consistent, reproducible assessment of the safety of an intervention is critically important in clinical care, research and safety surveillance.—In a multicentric, multinational observational study, severity assessment of neonatal AEs with the INC NAESS resulted in good interobserver agreement. This also highlights the need for INC NAESS user training, the need for more standardized safety data collection methods and the need for further expansion of the number of specific adverse events covered by INC NAESS.

## 5 Future perspectives

Based on the collective expertise acquired during development and validation of the NAESS tool and the subsequent stakeholders opinions and impact assessment, we want to reflect on future perspectives on the NAESS tool. We also wish to address more reliable assessment of AE severity to support regulatory decisions, and to enable weighted comparison between different interventions and facilitate shared decision making. These future perspectives relate to accessibility to the tool and teaching abilities, further development of the NAESS tool by adding AEs not yet covered, and consider additional standardization of AE case report forms to further improve inter-rater variability.

Related to access, a detailed description of the generic and all 35 specific AEs is provided in the initial paper and one of its supplements (7). We explicitly mention this as interested parties have contacted us to retrieve this specific information. This is perhaps because its access (under “INC Terminology”), through the Thesaurus of the US National Cancer Institute, linked to the Medical Dictionary for Regulatory Activities (MedDRA) is of relevance for regulatory science, but makes the information perhaps somewhat more technical (9). For training purposes, we provided a PowerPoint presentation as supplemental material to the prospective validation study paper (11). The INC is working to create a web-based, polished version of the NAESS training tool which will be made available for all interested parties.

Related to adding new specific AE tables, The first 35 AEs were selected as part of the modified Delphi procedure, while the NAESS needs further stepwise development. An INC work group has been tasked with the development of references for lab values. This is because lab values in other populations are commonly assessed during severity scoring. However, there is no standards for reporting laboratory values in neonates and the publication quality of laboratory values in clinical studies in neonates turned out to be sparse, not systematic and incomplete (8). Once a standard reference range for lab values is available, then we intend to apply this standard to large pooled real world datasets of laboratory values collected by the INC. To involve the users in the prioritization, requests to add a new AE or to adapt some of the scales in this NAEES document can therefore be filed through a Thesaurus link (https://ncitermform.nci.nih.gov/ncitermform/?dictionary=NCI%20Thesaurus). We encourage users to provide their input through this link, or otherwise, simply to reach out to one of the corresponding authors of this paper, or the NAESS development and validation efforts (7, 10, 11).

Finally, in both the retrospective and the prospective studies, we hypothesize that the most ideal reliability would be obtained in a setting where structured case report forms are available. This would not only standardize the language used to determine severity, but also how data are collected. Having standardized data collection tools, ensures that the assessors responsible for the (severity) grading of AE's are exposed to a structured version of the “noisy” clinical reality. It is reasonable to state that a severity assessment can only be as good as the quality of the information and observations collected. Further progress in standardization of safety information in newborns can be achieved by developing new digital tools that support clinical data extraction from the electronic health record as an approach to structured reporting. Obviously, this would preferably be achieved without raising the administrative burden, while still ensuring that all elements necessary for severity assessment are available, and accurate (reflecting the data as assessed by the clinical research team) (10, 11).

In conclusion, the INC developed a first version of the NAESS tool. This version underwent subsequently validation in both retro- and prospective trial design to assess its applicability and impact on the inter-observer variability. Since then, regulators, sponsors and academic researchers reported on the use of the NAESS in regulatory documents, trial protocols and study reports. We therefore reported on the trajectory and current status and impact of the NAESS score, on how different stakeholders within INC assessed its relevance, and on perspectives to further develop this tool. These future perspective relate to accessibility to the tool and teaching abilities, further development of the NAESS tool by adding specific AEs not yet covered, and on the idea to provide additional standardization of AE case record forms to further improve inter-rater variability. We hope that all stakeholders that are passionate about neonatal clinical trials and drug development will learn more about the NAESS tool and work to incorporate it into their study protocols, to improve efficiency and success of neonatal trials.

## Data Availability

The original contributions presented in the study are included in the article/Supplementary Material, further inquiries can be directed to the corresponding author.
